# Supplementary material for: Evaluation of PC‐ISO for customized, 3D printed, gynecologic 192Ir HDR brachytherapy applicators
Source: J Appl Clin Med Phys. 2015 Jan 8;16(1):246–53. doi: 10.1120/jacmp.v16i1.5168 (PMC5689973; doi:10.1120/jacmp.v16i1.5168)
Supplement: Supplementary file 1 — Supplementary Material [file ACM2-16-246-s001.pdf]

Evaluation of PC-ISO for Customized, 3D Printed, Gynecologic ^{192}Ir HDR Brachytherapy Applicators

Purpose: To evaluate the radiation attenuation properties of PC-ISO, a bio-compatible, sterilizable 3D printing material, and its suitability for customized, single-use gynecologic (GYN) brachytherapy applicators that have the potential for accurate guiding of seeds through linear and curved internal channels.

Method: A custom radiochromic film dosimetry apparatus was 3D-printed in PC-ISO with a single catheter channel and a slit to hold a radiochromic segment. The geometry of the apparatus was designed specifically to test geometry pertinent for use of this material in a clinical setting. A brachytherapy dose plan was computed to deliver a cylindrical dose distribution to the film. The dose plan used an ^{192}Ir source and was normalized to 1500 cGy at 1 cm from the channel. The material was evaluated by comparing the film exposure to an identical test done in water. The Hounsfield unit (HU) distributions were computed from a CT scan of the apparatus and compared to the HU distribution of water and the HU distribution of a commercial GYN cylinder applicator.

Results: The dose-depth curve of PC-ISO as measured by the radio chromic film was within 1% of water between 1 cm and 6 cm from the channel. The mean HU was -10 for PC-ISO and -1 for water. As expected, the honeycombed structure of PC-ISO resulting from the 3D printing process created a spread of HU values, but the mean was comparable to water.

Conclusions: PC-ISO is sufficiently water equivalent to be compatible with our HDR brachytherapy planning system and clinical workflow, and therefore it is suitable for creating custom GYN brachytherapy applicators. This procedure has been implemented in our clinic and custom GYN applicators made of commercially available PC-ISO are used when doing so can improve the patient's treatment.

Keywords: brachytherapy, 3D printing, custom applicators, sterilization, radiochromic

I. INTRODUCTION

Gynecologic (GYN) brachytherapy applicators come in a variety of shapes and sizes to accommodate different patient scenarios. However, there is little opportunity to customize the shape of these applicators and their internal structure to the needs of each patient. As a consequence, a fixed applicator might fit too loosely, which allows movement between scanning and treatment, and therefore increases dose uncertainty; it might fit too tightly, which can cause patient discomfort; or it might require extra interstitial catheters to ensure that dose objectives can be met.

Rapid prototyping, or 3D printing, has the potential to address the customization limitation of current GYN brachytherapy applicators. With 3D printing, it is possible to construct conformal applicators with customized channels [1]. There is currently a wide range of printing materials available for this purpose. However, to be suitable for clinical use, the material must be compatible with the brachytherapy workflow. Specifically, it must be biocompatible, sterilizable, free of CT scanning artifacts, and have similar dose attenuation properties as water (the medium assumed by brachytherapy planning systems using the AAPM Task Group 43 formalism).

The purpose of this study is to evaluate PC-ISO (Stratasys, Eden Prairie, MN), a biocompatible, thermoplastic, 3D printing material, for use in printing custom, single-use GYN brachytherapy applicators. Previous studies have shown that PC-ISO can be sterilized in multiple ways [2], including STERRAD (Advanced Sterilization Products, Irvine, CA), which is the preferred sterilization method at our institution.

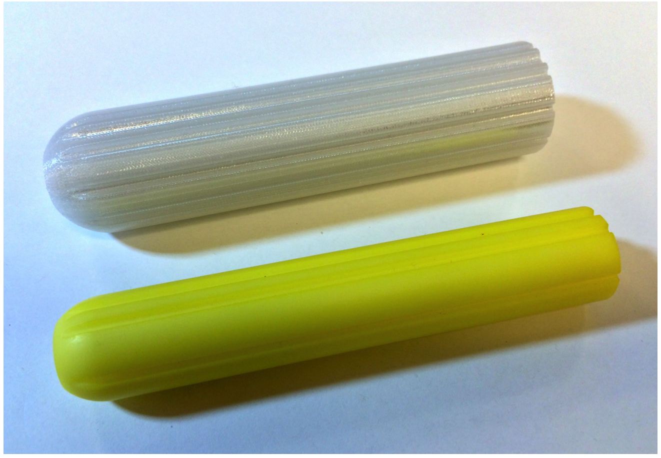

FIG. 1. 3D printing allows physicists and physicians to customize the size and shape of brachytherapy applicators to improve treatment. Shown above is a 3D printed applicator made of PC-ISO (white, top). This applicator was designed to fit a patient with a very wide vaginal canal, which was too large for the largest commercial applicator of the same type at our clinic (yellow, bottom).

This study evaluates the radiation properties of PC-ISO as a material for customized, single-use, GYN brachytherapy applicators. The evaluation is made using comparisons of CT scans, dose-depth curves for PC-ISO and water, and using geometry that is within the scope of a typical clinical procedure. Although this study focuses on evaluating PC-ISO, the same tests can be used to evaluate other materials for brachytherapy. Figure 1 shows an example of a customized GYN cylinder appli-

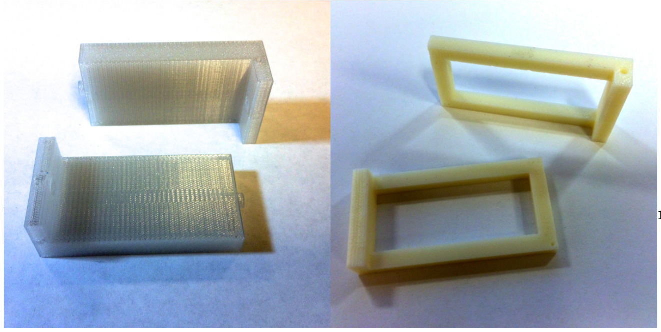

FIG. 2. A set of testing apparati were designed and 3D printed for this study to measure the depth-dose curve for ^{192}Ir. The apparati held a piece of radiochromic film and an endobronchial brachytherapy catheter. The left picture shows the testing apparatus printed in PC-ISO, and the picture on the right shows control apparatus used to suspend a piece of radiochromic film in water. The apparati were scanned in a helical CT to compute the Hounsfield unit distribution.

cator printed in PC-ISO next to a commercial applicator of the same type.

II. BACKGROUND

There are currently many medical applications of 3D printing in development [3–12]. This surge of interest includes medical modeling for maxillofacial surgical management [6, 13], bone reconstructions [8, 14], and oral surgeries [15]. The precision of 3D printers has been closely evaluated for medical applications with several studies confirming high levels of precision [16–18]. The Fortus 400mc (Stratasys) used in this study has a resolution for PC-ISO of 0.178 mm.

There is evidence of interest in 3D printing for radiotherapy applications [5, 9, 10, 16, 19, 20] and specifically in brachytherapy [4, 21–24]. There is even interest in using 3D printing to construct custom GYN applicators [3, 25]. However, to our knowledge, a 3D printed, GYN applicator has not been used to treat a patient yet.

Manufacturers have supported medical interests in 3D printing by introducing printing materials that pass the International Standard ISO-10993 as well as the United States Pharmacopeia standards for biocompatibility [26]. PC-ISO is both USP Class VI approved and ISO-10993-1 rated. The material is also sterilizable and has high flexural and tensile strength properties that have made it a common choice for many medical applications [27–29]. For example, PC-ISO has been explored for use in ankle-foot orthoses [14], lumbar cages [30], and bone screw linking devices [31].

III. METHODS AND MATERIALS

To evaluate PC-ISO, a custom testing apparatus was designed in CAD. This apparatus is shown in Figure 2 (left). The apparatus consisted of a pair of identical L-shaped blocks designed to snap together. Each block contained a single, straight channel 0.2 cm in diameter, which tightly held a 6F endobronchial brachytherapy catheter (Nucletron, Sunnyvale, CA). When snapped together, the blocks held a 3 cm by 6 cm radiochromic film segment in a 6 cm long shallow gap between the blocks. The blocks were 1 cm thick on each side of the film to provide side scatter on the scale of a typical brachytherapy applicator radius. The 6 cm side of the film was radial to the channel, and 3 cm side of the film was 0.25 cm away from the central axis of the channel.

The choice of a 3 cm diameter was used because it is a characteristic dimension of the typical cylinder applicator used in the clinic. The geometry of the apparatus was designed to be relevant to the geometry of applicators used in a clinical setting since the focus of this study was on the validation of PC-ISO for use in a brachytherapy clinical setting.

A nearly identical control apparatus was designed to leave most of the surface area of the film exposed. This apparatus was used to perform a control experiment in water. This apparatus is also shown in Figure 2 (right).

The testing apparatus was printed in PC-ISO using a Fortus 400mc (Stratasys), and the control apparatus was printed in ABS plastic using a uPrint Plus (3D Systems, San Francisco, CA). The stereolithography (STL) files for the testing and control blocks are available from the authors upon request.

A size 6 French endobronchial brachytherapy catheter was placed in the testing apparatus. The opposite channel was left empty. There was 3 cm of channel length inside the apparatus, which allowed for 13 dwell positions spaced 0.25 mm apart. Figure 3 shows the experimental setup. A dose plan with a cylindrical dose distribution was designed with equal dwell time at each of the 13 positions. The time was normalized to deliver 1500 cGy at 1 cm from the center of the channel in water. Figure 4 shows the isodose lines in Oncentra (Nucletron, Sunnyvale, CA) within the apparatus as would result from the designed dose plan with a ^{192}Ir source.

A 3 cm $\times$ 6 cm radiochromic film segment was placed between the two halves of the apparatus and the halves were snapped together. The entire apparatus was submerged in water, and the dose plan was delivered to the film using a microSelectron V2 digital afterloader. The same test was repeated on the control apparatus directly afterwards. The films were allowed to develop for 24 hours after exposure. Then they were processed to find the dose-depth curve.

A helical CT was used to scan the PC-ISO testing apparatus, a Nucletron cylinder applicator routinely used in the clinic, and a cup of water. The distribution of Hounsfield units (HU) was extracted for each from the

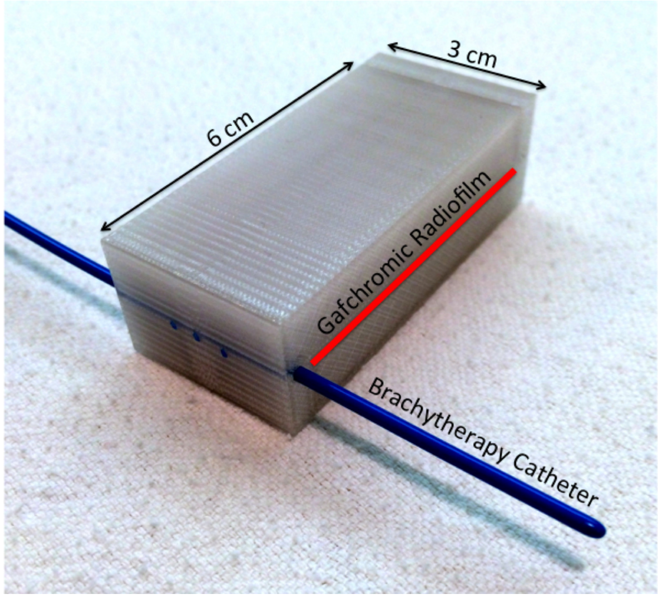

FIG. 3. The test apparatus with a size 6 French endobronchial brachytherapy catheter was inserted into one of the end channels. The PC-ISO and control apparati were suspended in water before the dose was delivered.

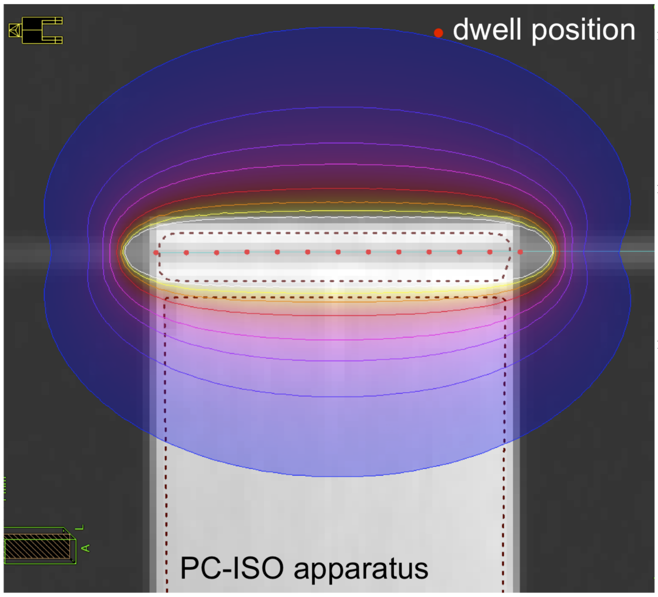

FIG. 4. A dose plan was to deliver 1500 cGy at 1 cm from the center of the catheter channel in water. The dwell positions and radial dose distribution for the radiochromic film study are represented using the TG-43 dose calculation formalism in water. The films were developed for 24 hours after exposure before they were scanned to find the dose-depth curve.

DICOM RT files.

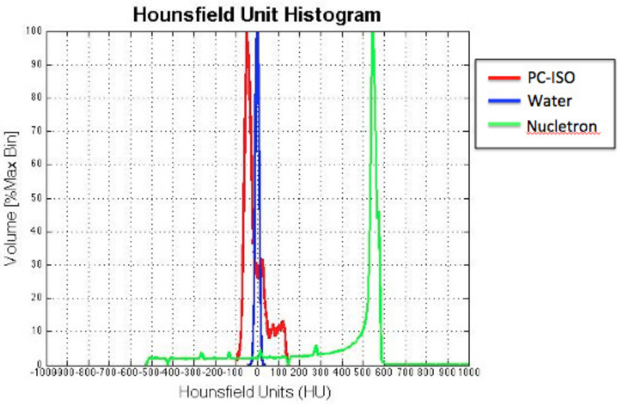

FIG. 5. Shown above is the distribution of Hounsfield units (HU) inside the PC-ISO apparatus (red), a Nucletron cylinder applicator (green), and a cup of water (blue). The mean was −1 HU for water, −10 HU for PC-ISO, and +524 for the Nucletron applicator. The mean HU value for the PC-ISO testing apparatus is closer to water than air (−1000 HU) or bone (+1000 HU) and more water equivalent than the Nucletron cylinder.

IV. RESULTS

There were no visible CT artifacts inside the testing apparatus. The distribution of the Hounsfield units (HU) inside the apparatus is shown in Figure 5. The mean Hounsfield unit was −1 HU for water and −10 HU for PC-ISO. This mean HU value is closer to water than air (−1000 HU) or bone (+1000 HU). The mean HU for the testing apparatus was more equivalent to water than the mean HU for the Nucletron cylinder applicator, which had a mean of +524 HU.

The percent dose depth for the testing apparatus (PC-ISO) and the control apparatus (water) is shown in Figure 6. The two curves are within 1% of each other between 1 cm and 6 cm from the channel. Doses closer than 1 cm were excluded because that region of the film was over-saturated.

V. DISCUSSION

To be compatible with current dose planning systems, PC-ISO should be radiologically equivalent to water within the energy range of interest, which for ^{192}Ir is approximately 10^2 keV with an average gamma emission energy of 380 keV. The results showed a 1% difference in dose attenuation over the range of interest, which for brachytherapy is not a significant source of error compared to other sources of error such as catheter movement and contouring uncertainty. The dose attenuation results are corroborated by the HU distribution, which did not show any very-high density regions in the printed medium that could effect the dose attenuation in an un-

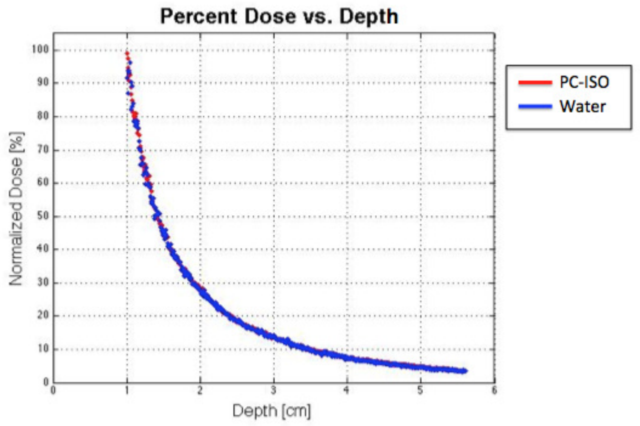

FIG. 6. The percent dose depth from the radiochromic film test for the PC-ISO testing apparatus (red) and the control apparatus (blue), which is water equivalent. The two curves were within 1% of each other between 1 cm and 6 cm, showing that the TG-43 planning system, which assumes a water medium, can be used as normal.

expected way. The spread seen for PC-ISO (Figure 5) is due to the honeycomb internal structure characteristic of 3D printing, which creates small regular-patterned regions of higher (material) and lower (air) density. The evaluation of PC-ISO's radiation properties along with previous studies of its mechanical properties and sterilization [14, 26–31] made us confident that it was suitable for clinical use.

Since the conclusion of these tests, we have offered customized PC-ISO to patients in cases where the physician felt it would improve their treatment. We printed a 2.75 cm diameter segmented cylinder, which is between two standard sizes (2.5 cm and 3.0 cm) from our vendor. We printed a 2.75 and a 3.5 cm diameter solid cylindrical applicator with internal channels. These applicators were custom built for each patient from measurements taken during examination. All PC-ISO applicators were sterilized using the STERRAD procedure before implantation. A CT scan of a custom-built PC-ISO cylinder applicator implanted in a patient prior to treatment is shown in Figure 7. The PC-ISO applicator is contoured on the scan but is difficult to see because of its near water-equivalency. The Nucletron tandem used during the procedure inserted in the center of the printed cylinder is clearly visible at the center of the applicator.

PC-ISO applicators are virtually tissue-equivalent under CT scan – more tissue-equivalent than commercial applicators at our clinic. This level of tissue equivalence can make it difficult to find the boundary of the applicator during segmentation, especially at the tip of the applicator where the surface is curved. To address this issue, it may be possible to cover the applicators in a radio-opaque dye and condom before insertion.

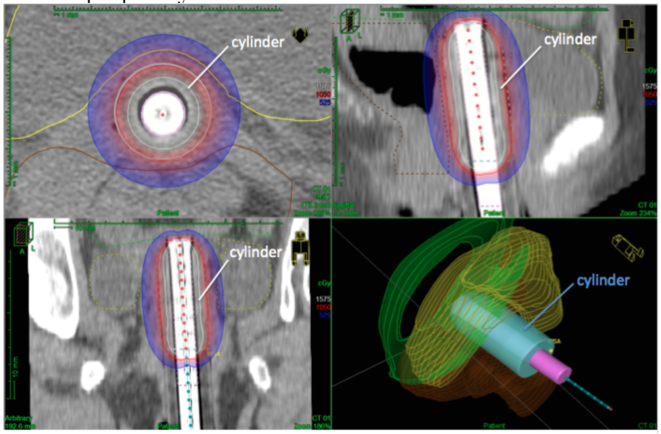

FIG. 7. After the conclusion of the tests outlined in this study, we printed several GYN applicators in PC-ISO when a custom built applicator would improve treatment options. The applicators were designed specifically for each patient from measurements taken during examination and were sterilized using the STERRAD procedure before implantation and treatment. A segmented PC-ISO cylinder applicator with a custom 2.75 cm diameter is shown implanted and contoured in the patient during the dose planning process in axial (top left), sagittal (top right), and coronal (bottom left) view. The Nucletron tandem (bright white) is approximately 1/3 the diameter of the full applicator. Bottom right is a 3D representation of the contoured organs, applicator (blue), and tandem (purple) with the catheter visible.

VI. CONCLUSION

PC-ISO is a readily available material for 3D printing that is FDA approved for temporary implants. In this study it was evaluated for use in a brachytherapy environment. It was shown that PC-ISO has sufficiently equivalent dose attenuation properties to water at ^{192}Ir energies to be compatible with the brachytherapy planning system and workflow. It also does not produce CT artifacts. Given these results, we printed several customized cylinders and used these cylinders on patients when it would improve their treatment. While 3D printers with the capability to print in FDA-approved materials are currently on the order of $100,000, clinics wishing to implement this technique can outsource the printing of their customized designs to printing vendors.

VII. ACKNOWLEDGEMENTS

We thank the Qualcomm Undergraduate Experiences in Science and Technology (QUEST) program for providing funding and resources for this project. We also thank Serena Scott, Ph. D, for her help designing the applicators.

[1] A. Garg, S. Patil, T. Siauw, J. A. M. Cunha, I. Hsu, P. Abbeel, J. Pouliot, K. Goldberg *et al.*, "An algorithm for computing customized 3d printed implants with curvature constrained channels for enhancing intracavitary brachytherapy radiation delivery," in *Automation Science and Engineering (CASE), 2013 IEEE International Conference on.* IEEE, 2013, pp. 466–473.

[2] M. Perez, M. Block, D. Espalin, R. Winker, T. Hoppe, F. Medina, and R. Wicker, "Sterilization of fdm-manufactured parts," *Twenty Third Annual International Solid Freeform Fabrication Symposium - An Additive Manufacturing Conference*, pp. 285–296, 2012.

[3] M. Albano, I. Dumas, and C. Haie-Meder, "Brachytherapy at the institut gustave-roussy: personalized vaginal mould applicator: technical modification and improvement," *Cancer Radiother*, vol. 12, pp. 822–6, 2008.

[4] M. Poulsen, C. Lindsay, T. Sullivan, and P. DUrso, "Stereolithographic modelling as an aid to orbital brachytherapy," *International Journal of Radiation Oncology* Biology* Physics*, vol. 44, no. 3, pp. 731–735, 1999.

[5] C. Zemnick, S. A. Woodhouse, R. M. Gewanter, M. Raphael, and J. D. Piro, "Rapid prototyping technique for creating a radiation shield," *The Journal of Prosthetic Dentistry*, vol. 97, no. 4, pp. 236–241, 2007.

[6] L. K. Chow and L. K. Cheung, "The usefulness of stereomodels in maxillofacial surgical management," *Journal of Oral and Maxillofacial Surgery*, vol. 65, no. 11, pp. 2260–2268, 2007.

[7] H. Dang and Z. Chen, "Integration of cbct and a skull base drilling robot," 2011.

[8] A. Cohen, A. Laviv, P. Berman, R. Nashef, and J. Abu-Tair, "Mandibular reconstruction using stereolithographic 3-dimensional printing modeling technology," *Oral Surgery, Oral Medicine, Oral Pathology, Oral Radiology, and Endodontology*, vol. 108, no. 5, pp. 661–666, 2009.

[9] B. Guix, F. Finestres, J.-I. Tello, C. Palma, A. Martinez, J.-R. Guix, and R. Guix, "Treatment of skin carcinomas of the face by high-dose-rate brachytherapy and custom-made surface molds," *International Journal of Radiation Oncology* Biology* Physics*, vol. 47, no. 1, pp. 95–102, 2000.

[10] K. Obinata, K. Ohmori, H. Shirato, and M. Nakamura, "Experience of high-dose-rate brachytherapy for head and neck cancer treated by a customized intraoral mold technique," *Radiation Medicine*, vol. 25, no. 4, pp. 181–186, 2007.

[11] E. Bassoli, A. Gatto, L. Iuliano, and M. G. Violante, "3d printing technique applied to rapid casting," *Rapid Prototyping Journal*, vol. 13, no. 3, pp. 148–155, 2007.

[12] F. Rengier, A. Mehndiratta, H. von Tengg-Kobligk, C. Zechmann, R. Unterhinninghofen, H.-U. Kauczor, and F. Giesel, "3d printing based on imaging data: review of medical applications," *International journal of computer assisted radiology and surgery*, vol. 5, no. 4, pp. 335–341, 2010.

[13] M. Anchieta, M. Quaresma, and F. de Salles, "Rapid prototyping applied to maxillofacial surgery," *Advanced Applications of Rapid Prototyping Technology in Modern Engineering. Rijeka, Croatia: InTech*, pp. 153–72, 2011.

[14] E. S. Schrank, L. Hitch, K. Wallace, R. Moore, and S. J. Stanhope, "Assessment of a virtual functional prototyping process for the rapid manufacture of passive-dynamic ankle-foot orthoses," *Journal of biomechanical engineering*, vol. 135, no. 10, p. 101011, 2013.

[15] J. Winder, R. Bibb *et al.*, "Medical rapid prototyping technologies: state of the art and current limitations for application in oral and maxillofacial surgery." *Journal of oral and maxillofacial surgery: official journal of the American Association of Oral and Maxillofacial Surgeons*, vol. 63, no. 7, p. 1006, 2005.

[16] J. Winder, "Medical rapid prototyping and 3d ct in the manufacture of custom made cranial titanium plates," *Journal of medical engineering & technology*, vol. 23, no. 1, pp. 26–28, 1999.

[17] J.-Y. Choi, J.-H. Choi, N.-K. Kim, Y. Kim, J.-K. Lee, M.-K. Kim, J.-H. Lee, and M.-J. Kim, "Analysis of errors in medical rapid prototyping models," *International journal of oral and maxillofacial surgery*, vol. 31, no. 1, pp. 23–32, 2002.

[18] P. Webb, "A review of rapid prototyping (rp) techniques in the medical and biomedical sector," *Journal of Medical Engineering & Technology*, vol. 24, no. 4, pp. 149–153, 2000.

[19] E. Schwaderer, A. Bode, W. Budach, C. Claussen, F. Dammann, T. Kaus, and P. Plinkert, "Soft-tissue stereolithographic model as an aid to brachytherapy," *medicamundi*, vol. 44, no. 1, pp. 48–51, 2000.

[20] T. Juang, P. Stauffer, D. Neuman, and J. Schlorff, "Multilayer conformal applicator for microwave heating and brachytherapy treatment of superficial tissue disease," *International journal of hyperthermia*, vol. 22, no. 7, pp. 527–544, 2006.

[21] A. Pompeu-Robinson, M. Kunz, C. Falkson, L. Schreiner, C. Joshi, and G. Fichtinger, "Immobilization and catheter guidance for breast brachytherapy," *International journal of computer assisted radiology and surgery*, vol. 7, no. 1, pp. 65–72, 2012.

[22] S. Mutic, P. W. Grigsby, D. A. Low, J. F. Dempsey, W. B. Harms, R. Laforest, W. R. Bosch, and T. R. Miller, "Pet-guided three-dimensional treatment planning of intracavitary gynecologic implants," *International Journal of Radiation Oncology* Biology* Physics*, vol. 52, no. 4, pp. 1104–1110, 2002.

[23] M. Maalej, C. Ben Ammar, L. Kochbati, H. Frikha, D. Hentati, W. Gargouri, and M. Besbes, "Brachytherapy for primary and recurrent nasopharyngeal carcinoma: treatment techniques and results," *Cancer/Radiothérapie*, vol. 11, no. 3, pp. 117–121, 2007.

[24] N. Makni, A. Iancu, P. Puech, S. Mordon, and N. Betrouni, "A morphological atlas of prostates zonal anatomy for construction of realistic digital and physical phantoms," *Prostate Cancer Imaging. Image Analysis and Image-Guided Interventions*, pp. 22–34, 2011.

[25] E. Wiebe, G. Thomas, L. Barbera, H. Easton, and A. Ravi, "Customized vaginal vault brachytherapy with ct imaging-derived applicator prototyping," *Cancer/Radiothérapie*, vol. 12, p. 822, 2008.

[26] J. M. Anderson and J. J. Langone, "Issues and perspectives on the biocompatibility and immunotoxicity evaluation of implanted controlled release systems," *Journal*

of controlled release, vol. 57, no. 2, pp. 107–113, 1999.

[27] L. Novakova-Marcincinova and J. Novak-Marcincin, "Experimental testing of materials used in fused deposition modeling rapid prototyping technology," *Advanced Materials Research*, vol. 740, pp. 597–602, 2013.

[28] S. Koo, B. A. Hargreaves, G. E. Gold, and J. L. Dragoo, "Fabrication of custom-shaped grafts for cartilage regeneration," *The International journal of artificial organs*, vol. 33, no. 10, p. 731, 2010.

[29] L. Hitch, K. Wallace, R. Moore, and S. J. Stanhope, "Assessment of a virtual functional prototyping process for the rapid manufacture of passive-dynamic ankle-foot orthoses."

[30] A. Aherwar, A. Singh, and A. Patnaik, "A review paper on rapid prototyping and rapid tooling techniques for fabrication of prosthetic socket," in *High Value Manufacturing: Advanced Research in Virtual and Rapid Prototyping: Proceedings of the 6th International Conference on Advanced Research in Virtual and Rapid Prototyping, Leiria, Portugal, 1-5 October, 2013.* CRC Press, 2013, p. 345.

[31] R. C. Groscurth and S. U. Groscurth, "Bone screw linking device," Sep. 15 2010, uS Patent App. 12/882,800.
