# Supplementary material for: Evaluation of PC‐ISO for customized, 3D printed, gynecologic 192Ir HDR brachytherapy applicators
Source: J Appl Clin Med Phys. 2015 Jan 8;16(1):246–53. doi: 10.1120/jacmp.v16i1.5168 (PMC5689973; doi:10.1120/jacmp.v16i1.5168)
Supplement: Supplementary file 2 — Supplementary Material [file ACM2-16-246-s002.doc]

**(**COMP: Special characters: xxby = sans-serif multiply; **)**

Cunha, et al.: PC-ISO for 3D printed brachytherapy applicators

**Evaluation of PC-ISO for customized, 3D printed, gynecologic ^192^Ir HDR brachytherapy applicators**

J. Adam M. Cunha,^1a^ Katherine Mellis,^1^ Rajni Sethi,^1^ Timmy Siauw,^1^ Atchar Sudhyadhom,^1^ Animesh Garg,^2^ Ken Goldberg,^1,2^ I-Chow Hsu,^1^ and Jean Pouliot^1^

*Radiation Oncology,^1^ University of California, San Francisco, CA; Electrical Engineering and Computer Science,^2^ University of California, Berkeley, CA, USA*

[Adam.Cunha@ucsf.edu](mailto:Adam.Cunha@ucsf.edu)

Received 25 June, 2014; accepted 30 September, 2014

^a^ Corresponding author: J. Adam M. Cunha, Radiation Oncology, University of California, 1600 Divisadero St., San Francisco, CA 94115, USA; phone: (415) 353 7031; fax: XXXX; email: [Adam.Cunha@ucsf.edu](mailto:Adam.Cunha@ucsf.edu)

[ABSTRACT]

The purpose of this study was to evaluate the radiation attenuation properties of PC-ISO, a commercially available, biocompatible, sterilizable 3D printing material, and its suitability for customized, single-use gynecologic (GYN) brachytherapy applicators that have the potential for accurate guiding of seeds through linear and curved internal channels. A custom radiochromic film dosimetry apparatus was 3D-printed in PC-ISO with a single catheter channel and a slit to hold a film segment. The apparatus was designed specifically to test geometry pertinent for use of this material in a clinical setting. A brachytherapy dose plan was computed to deliver a cylindrical dose distribution to the film. The dose plan used an ^192^Ir source and was normalized to 1500 cGy at 1 cm from the channel. The material was evaluated by comparing the film exposure to an identical test done in water. The Hounsfield unit (HU) distributions were computed from a CT scan of the apparatus and compared to the HU distribution of water and the HU distribution of a commercial GYN cylinder applicator. The dose depth curve of PC-ISO as measured by the radiochromic film was within 1% of water between 1 cm and 6 cm from the channel. The mean HU was -10 for PC-ISO and -1 for water. As expected, the honeycombed structure of the PC-ISO 3D printing process created a moderate spread of HU values, but the mean was comparable to water. PC-ISO is sufficiently water-equivalent to be compatible with our HDR brachytherapy planning system and clinical workflow and, therefore, it is suitable for creating custom GYN brachytherapy applicators. Our current clinical practice includes the use of custom GYN applicators made of commercially available PC-ISO when doing so can improve the patient’s treatment.

PACS number:

Key words: brachytherapy, 3D printing, custom applicators, sterilization, radiochromic film

# I. Introduction

Gynecologic (GYN) brachytherapy applicators come in a variety of shapes and sizes to accommodate different patient scenarios. However, there is little opportunity to customize the shape of these applicators and their internal structure to the needs of each patient. As a consequence, a fixed applicator might fit too loosely, which allows movement between scanning and treatment, and therefore increases dose uncertainty; it might fit too tightly, which can cause patient discomfort; or it might require extra interstitial catheters to ensure that dose objectives can be met.

Rapid prototyping, or 3D printing, can address the customization limitation of current GYN brachytherapy applicators. With 3D printing, it is possible to construct conformal applicators with customized channels.^(1)^ There is currently a wide range of printing materials available for this purpose. However, to be suitable for clinical use, the material must be compatible with the brachytherapy workflow. Specifically, it must be biocompatible, sterilizable, free of CT scanning artifacts, and have similar dose attenuation properties as water (the medium assumed by brachytherapy planning systems using the AAPM Task Group 43 formalism).

The purpose of this study is to evaluate PC-ISO (Stratasys, Eden Prairie, MN), a commercially available, biocompatible, thermoplastic, 3D printing material, for use in printing custom, single-use GYN brachytherapy applicators. PC-ISO is a polycarbonate (i.e., its molecular composition consists of polymers containing carbonate groups). Specifications of the material ( e.g., tensile strength, flexural modulus) can be found in the specifications sheet on the StrataSys webpage: [www.stratasys.com/materials/fdm/pc-iso](http://www.stratasys.com/materials/fdm/pc-iso). Previous studies have shown that PC-ISO can be sterilized in multiple ways,^(2)^ including STERRAD (Advanced Sterilization Products, Irvine, CA), which is the preferred sterilization method at our institution.

This study evaluates the radiation properties of PC-ISO as a material for customized, single-use, GYN brachytherapy applicators. The evaluation is made using comparisons of CT scans, dose depth curves for PC-ISO and water, and using geometry that is within the scope of a typical clinical procedure. Although this study focuses on evaluating PC-ISO, the same tests can be used to evaluate other materials for brachytherapy. Figure 1 shows an example of a customized GYN cylinder applicator printed in PC-ISO next to a commercial applicator of the same type from Alpha Omega Services, Inc (Bellflower, CA) that is routinely used in our clinic.

**A. Background**

There are currently many medical applications of 3D printing in development.^(3–12)^ This surge of interest includes medical modeling for maxillofacial surgical management,^(6,13)^ bone reconstructions,^(8,14)^ and oral surgeries.^(15)^ The precision of 3D printers has been closely evaluated for medical applications with several studies confirming high levels of precision.^(16–18)^ The Fortus 400mc (Stratasys) used in this study has a resolution for PC-ISO of 0.178 mm.

There is evidence of interest in 3D printing for radiotherapy applications^(5,9,10,16,19,20)^ and specifically in brachytherapy.^(4,21–24)^ There is even interest in using 3D printing to construct custom GYN applicators.^(3,25)^ However, to our knowledge, a 3D printed, GYN applicator has not been used as yet to treat a patient. Brachytherapy practitioners do have a long history of creating custom applicators uniquely fabricated to treat individual patients using common materials. This innovation continues today, especially in the application of head and neck brachytherapy where intrapatient anatomy varies considerably.^(9,10)^

Manufacturers have supported medical interests in 3D printing by introducing printing materials that pass the International Standard ISO-10993, as well as the United States Pharmacopeia (USP) standards for biocompatibility.^(26)^ PC-ISO is both USP Class VI approved and ISO-10993-1 rated. This FDA-approved material is commercially available, sterilizable, approved for temporary implants, and has high flexural and tensile strength properties that have made it a common choice for many medical applications.^(27–28,14)^ For example, PC-ISO has been explored for use in ankle-foot orthoses,^(14)^ lumbar cages,^(29)^ and bone screw linking devices.^(30)^

# II. Materials and Methods

To evaluate PC-ISO, a custom testing apparatus was designed in CAD. This apparatus is shown in Fig. 2 (left). The apparatus consisted of a pair of identical L-shaped apparati designed to snap together. Each half contained a single, straight channel 2 mm in diameter, which snugly held a 6F endobronchial brachytherapy catheter (Nucletron, Sunnyvale, CA). When snapped together (Fig. 3), the halves held a 3 cm by 6 cm radiochromic film segment in a 6 cm long shallow gap between the assembled apparatus. The assembled apparatus was 1 cm thick on each side of the film which, when the apparatus is submerged in a water bath, provided scatter conditions on the scale of a typical vaginal brachytherapy applicator radius. The 6 cm side of the film was radial to the channel, and the 3 cm side of the film was 0.25 cm from the central axis of the channel.

A 2 cm xxby 3 cm geometry was used because it represents a characteristic dimension of the typical cylinder applicator used in the clinic. The apparatus was designed to be relevant to the geometry of applicators used in a clinical setting since the focus of this study was on the validation of PC-ISO for use in a brachytherapy clinical setting.

A nearly identical control apparatus was designed to leave most of the surface area of the film exposed. This apparatus was used to perform a control experiment in water. This apparatus is also shown in Fig. 2 (right). The testing apparatus was printed in PC-ISO using a Fortus 400mc , and the control apparatus was printed in ABS plastic using a uPrint Plus (3D Systems, San Francisco, CA). ABS (acrylonitrile butadiene styrene) plastic is a part of the terpolymers family of thermoplastics that are made of three different monomers, acrylonitrile, butadiene, and styrene, and was first developed in the early 1950s; it has been one of the first material substrates for 3D printers. It is not approved per ISO-10993-1 as a biocompatible material, but recently has been used to make tissue-equivalent phantoms for IMRT QA.^(31)^ The stereolithography (STL) files for the testing and control apparati are available from the authors upon request.

A size 6-French endobronchial brachytherapy catheter was placed in the testing apparatus. The opposite channel was left empty. There was 3 cm of channel length inside the apparatus, which allowed for 13 dwell positions spaced 0.25 cm apart. Figure 3 shows the experimental setup. A dose plan with a cylindrical dose distribution was designed with equal dwell time at each of the 13 positions. The time was normalized to deliver 1500 cGy at 1 cm radially from the center dwell position in water. Figure 4 shows the isodose lines within the apparatus as would result from the designed dose plan with an ^192^Ir source and using the TG-43 dose calculation formalism, as calculated in the Nucletron Oncentra TPS^(32)^ (Nucletron BV, Veenendaal, The Netherlands).

A 3 cm by 6 cm radiochromic film (GAFCHROMIC EBT2) (International Specialty Products, Wayne, NJ) segment was placed between the two apparatus halves and the halves were snapped together. The entire apparatus was submerged in a water tank, and the dose plan was delivered to the film using a microSelectron V2 digital afterloader (Nucletron BV). The same test was repeated on the control apparatus directly afterwards. The films were allowed to self develop for 24 hrs after exposure. Then they were scanned at 300 dpi using an Epson Expression 10000XL scanner (US Epson, Long Beach, CA) to create a H&D that was used to extract the dose depth curve (following conversion to dose).

A helical Siemans Sensation Open CT (120 kVp, automatic tube current modulation, 1 mm slice thickness) (Siemens Medical Solutions, Malvern, PA) was used to scan the PC-ISO testing apparatus, an Alpha Omega cylinder applicator routinely used in the clinic (part number: AO#7260), and a cup of water. The distribution of Hounsfield units (HU) was extracted for each from the DICOM RT files.

# III. Results

The 2.0 mm catheter channel diameter in the apparati resulted in a snug fit of the 1.9 mm catheter (6-French) in the channel. The 0.1 mm tolerance between the catheter and channel results in a sliding fit with no internal movement. The catheter insertion into the channel required gentle pressure. Static friction held the catheter in place once inserted. Thus for the printing of these specific apparati, the ± 0.178 mm resolution of the printer resulted in a channel diameter smaller than the CAD design, but within the 0.1 mm tolerance of the design.

There were no visible CT artifacts inside the testing apparatus. The distribution of the Hounsfield units (HU) inside the apparatus is shown in Fig. 5. The mean Hounsfield unit was -1 HU for water and 10 HU for PC-ISO. This mean HU value is closer to water than air (−1000 HU) or bone (+1000 HU). The mean HU for the testing apparatus was more equivalent to water than the mean HU for the Alpha Omega cylinder applicator, which had a mean of +524 HU.

The percent dose depth for the testing apparatus (PC-ISO) and the control apparatus (water) is shown in Fig. 6. The two curves are within 1% of each other between 1 cm and 6 cm from the channel. Doses closer than 1 cm were excluded because that region of the film was oversaturated.

**IV. DISCUSSION**

To be compatible with current dose planning systems, PC-ISO should be radiologically equivalent to water within the energy range of interest, which for ^192^Ir is approximately 10^2^ keV, with an average gamma emission energy of 380 keV. The results showed a 1% difference in dose attenuation over the range of interest, which for brachytherapy is not a significant source of error compared to other sources of error such as catheter movement and contouring uncertainty. The dose attenuation results are corroborated by the HU distribution, which did not show any regions of very high density in the printed medium that could adversely affect the otherwise homogeneous dose attenuation in an unexpected way. The spread in HU seen for PC-ISO (Fig. 5) is likely due to the honeycomb internal structure characteristic of 3D printing, which creates small regular-patterned regions of higher (material) and lower (air) density. The current evaluation of PC-ISO's radiation properties, along with previous studies of its mechanical properties and sterilization,^(14,26-30)^ made us confident that it was suitable for clinical use.

Since the conclusion of these tests, we have created customized PC-ISO applicators for patients in cases where the physician felt it would improve their treatment. We have printed a 2.75 cm and a 3.25 cm diameter segmented cylinder — similar to Nucletron’s Vaginal CT/MR applicator set (part #101.001), but between the standard sizes of 2.0 cm, 2.5 cm, 3.0 cm, and 3.5 cm. These cylinders used the original vaginal tube (part #101.002) from the Nucletron Vagnial CT/MR applicator set, but the four cylinder segments (parts #101.007 – #101.010) were replaced with 3D-printed, PC-ISO components. In addition to the channel for the central vaginal tube, the cylinder segments had 6 internal channels similar to the Nucletron Miami applicator set (part #085.210).

We also developed another design, based on the Alpha Omega applicator, AO#7260, (yellow cylinder, bottom Fig. 1). The Alpha Omega applicator is a single cylindrical piece with a central channel for a uterine tandem and 6 surface channels on the circumference. (The surface channels are grooves along the length of the cylinder to allow for brachytherapy catheters to be places directly on the surface of the applicator.) A patient presented for whom a larger diameter would be beneficial. However, because this applicator is available in the 2.5 cm diameter size only, a custom 3.5 cm diameter cylinder was designed which incorporated 10 circumferential channels (Fig. 1 top) and 1 central channel (10+1). The circumferential distance between each surface channel was approximately 1 cm (11 mm), which was desirable to avoid generating dose hotspots. A similar 10+1 channel applicator was also designed with a 3.0 cm diameter.

Each aforementioned applicator was designed according to measurements taken during examination. The 2 mm channels in our testing apparatus produced a tight fit for 6F catheters. Therefore, the custom applicators we used clinically were designed with 2.1 mm diameter channels. This allowed for easier insertion of the catheters with no resistance. All PC-ISO applicators were sterilized using the STERRAD procedure before implantation. The sterile cylinders were brought to the operating room (OR) along with the interstitial needles.

For each case the applicator and catheters were inserted into the patient under local anesthesia. After insertion, our standard HDR brachytherapy planning protocol was followed with no additional steps due to having a custom applicator: a CT scan was obtained; the images were imported into the Nucletron Oncentra treatment planning system where a plan was generated using the IPSA optimization engine; and the plan was delivered. The applicators were removed without issues after all fractions were delivered.

CT scans of a patient implanted with the 2.75 cm custom-built PC-ISO cylinder applicator is shown in Fig. 7. Isodose lines are visible (red = 100% Rx, blue = 50% Rx, white = 150% Rx). The PC-ISO applicator is contoured on the scan, but is difficult to see because of its near water equivalency. The Nucletron tandem used during the procedure inserted in the center of the printed cylinder is clearly visible at the center of the applicator.

PC-ISO applicators are mostly tissue-equivalent under CT scan — more tissue-equivalent than commercial applicators at our clinic. This level of tissue equivalence can make it difficult to find the boundary of the applicator during contouring, especially at the tip of the applicator where the surface is curved. To address this issue, it may be possible to cover the applicators in a radiopaque dye and condom before insertion.

# V. Conclusions

PC-ISO is a readily available material for 3D printing with FDA-approved biocompatibility for temporary implants in the body. In this study it was evaluated for use in a brachytherapy environment. It was shown that PC-ISO has sufficiently equivalent dose attenuation properties to water at ^192^Ir energies to be compatible with the brachytherapy planning system and workflow. It also does not produce CT artifacts. Given these results, we printed several customized cylinders and used these cylinders on patients when it would improve their treatment. While 3D printers with the capability to print in FDA-approved materials are currently on the order of $100,000, clinics can design and create custom, PC-ISO applicators without a 3D printer by outsourcing the printing to vendors.

# Acknowledgments

We thank the Qualcomm Undergraduate Experiences in Science and Technology (QUEST) program for providing funding and resources for this project. We also thank Serena Scott, Ph.D, for her help designing the applicators.

# References

1. Garg A, Patil S, Siauw T, et al. An algorithm for computing customized 3D printed implants with curvature constrained channels for enhancing intracavitary brachytherapy radiation delivery. In: 2013 IEEE International Conference on Automation Science and Engineering (CASE). Piscataway, NJ: IEEE, 2013. p. 466–73.

2. Perez M, Block M, Espalin D, et al. Sterilization of FDM-manufactured parts. Presented at the Twenty-third Annual International Solid Freeform Fabrication Symposium – An Additive Manufacturing Conference, 6-8 August 2011. Austin, TX: Laboratory for Solid Freeform Fabrication; 2012.

3. Albano M, Dumas I, Haie-Meder C. [Brachytherapy at the Institut Gustave-Roussy: personalized vaginal mould applicator: technical modification and improvement] [article in French]. Cancer Radiother. 2008;12(8):822–26.

4. Poulsen M, Lindsay C, Sullivan T, DUrso P. Stereolithographic modelling as an aid to orbital brachytherapy. Int J Radiat Oncol Biol Phys. 1999;44(3):731–35.

5. Zemnick C, Woodhouse SA, Gewanter RM, Raphael M, Piro JD. Rapid prototyping technique for creating a radiation shield. J Prosthet Dent. 2007;97(4):236–41.

6. Chow LK and Cheung LK. The usefulness of stereomodels in maxillofacial surgical management. J Oral Maxillofac Surg. 2007;65(11):2260–68.

7. Novakova-Marcincinova L and Novak-Marcincin J. Experimental testing of materials used in fused deposition modeling rapid prototyping technology. Adv Mat Res. 2013;740:597–602.

8. Cohen A, Laviv A, Berman P, Nashef R, Abu-Tair J. Mandibular reconstruction using stereolithographic 3-dimensional printing modeling technology. Oral Surg Oral Med Oral Pathol Oral Radiol Endod. 2009;108(5):661–66.

9. Guix B, Finestres F, Tello JI, et al. Treatment of skin carcinomas of the face by high-dose-rate brachytherapy and custom-made surface molds. Int J Radiat Oncol Biol Phys. 2000;47(1):95–102.

10. Obinata K, Ohmori K, Shirato H, Nakamura M. Experience of high-dose-rate brachytherapy for head and neck cancer treated by a customized intraoral mold technique. Radiat Med. 2007;25(4):181–86.

11. Bassoli E, Gatto A, Iuliano L, Violante MG. 3D printing technique applied to rapid casting. Rapid Prototyping J. 2007;13(3):148–55.

12. Rengier F, Mehndiratta A, von Tengg-Kobligk H, et al. 3D printing based on imaging data: review of medical applications. Int J Comput Assist Radiol Surg. 2010;5(4):335–41.

13. Anchieta M, Quaresma M, de Salles F. Rapid prototyping applied to maxillofacial surgery. Advanced Applications of Rapid Prototyping Technology in Modern Engineering. InTech ([www.intechopen.com](http://www.intechopen.com/)). 2011:153–72.

14. Schrank ES, Hitch L, Wallace K, Moore R, Stanhope SJ. Assessment of a virtual functional prototyping process for the rapid manufacture of passive-dynamic ankle-foot orthoses. J Biomechan Eng. 2013;135(10):101011.

15. Winder J and Bibb R. Medical rapid prototyping technologies: state of the art and current limitations for application in oral and maxillofacial surgery. J Oral Maxillofac Surg. 2005;63(7):1006.

16. J. Winder, Cooke RS, Gray J, Fannin T, Fegan T. Medical rapid prototyping and 3D CT in the manufacture of custom made cranial titanium plates. J Med Eng Tech. 1999;23(1):26–28.

17. Choi JY, Choi JH, Kim NK, et al. Analysis of errors in medical rapid prototyping models. Int J Oral Maxillofac Surg. 2002;31(1):23–32.

18. Webb P. A review of rapid prototyping (RP) techniques in the medical and biomedical sector. J Med Eng Tech. 2000;24(4):149–53.

19. Schwaderer E, Bode A, Budach W, et al. Soft-tissue stereolithographic model as an aid to brachytherapy. Medicamundi. 2000;44(1):48–51.

20. Juang T, Stauffer P, Neuman D, Schlor J. Multilayer conformal applicator for microwave heating and brachytherapy treatment of superficial tissue disease. Int J Hyperthermia. 2006;22(7):527–44.

21. Pompeu-Robinson A, Kunz M, Falkson C, Schreiner L, Joshi C, Fichtinger G. Immobilization and catheter guidance for breast brachytherapy. Int J Comput Assist Radiol Surg. 2012;7(1):65–72.

22. Mutic S, Grigsby PW, Low DA, et al. PET-guided three-dimensional treatment planning of intracavitary gynecologic implants. Int J Radiat Oncol Biol Phys. 2002;52(4):1104–10.

23. Maalej M, Ben Ammar C, Kochbati L, et al. Brachytherapy for primary and recurrent nasopharyngeal carcinoma: treatment techniques and results. Cancer Radiother. 2007;11(3):117–21.

24. Makni N, Iancu A, Puech P, Mordon S, Betrouni N. A morphological atlas of prostates zonal anatomy for construction of realistic digital and physical phantoms. Prostate Cancer Imaging. 2011; LNCS 6963:22–34.

25. Wiebe E, Thomas G, Barbera L, Easton H, Ravi A. Customized vaginal vault brachytherapy with CT imaging-derived applicator prototyping. Int J Radiat Oncol Biol Phys. 2012;84(3):S870.

26. Anderson JM and Langone JJ. Issues and perspectives on the biocompatibility and immunotoxicity evaluation of implanted controlled release systems. J Control Release. 1999;57(2):107–13.

27. Novakova-Marcincinova L and Novak-Marcincin J. Experimental testing of materials used in fused deposition modeling rapid prototyping technology. Adv Mat Res. 2013;740:597–602.

28. Koo S, Hargreaves BA, Gold GE, Dragoo JL. Fabrication of custom-shaped grafts for cartilage regeneration. Int J Artificial Organs. 2010;33(10):731–37.

29. Aherwar A, Singh A, Patnaik A. A review paper on rapid prototyping and rapid tooling techniques for fabrication of prosthetic socket, Chapter 50. In: daSilva Bartolo PJ, Soares de Lemos AC, Pereira AM, et al., editors. High Value Manufacturing: Advanced Research in Virtual and Rapid Prototyping: Proceedings of the 6th International Conference on Advanced Research in Virtual and Rapid Prototyping. Leiria, Portugal, 1-5 October, 2013. London, UK: CRC Press; 2013.

30. Groscurth RC and Groscurth SU, inventors. Bone screw linking device. United States Patent App. 12/882,800. 2010 Sep. 15.

31. Kumar R, Sharma SD, Despande S, et al. Acrylonitrile Butadiene Styrene (ABS) plastic-based low cost tissue equivalent phantom for verification dosimetry in IMRT. J Appl Clin Med Phys. 2010;11(1):24–32.

32. Daskalov G, Löffler E, Williamson J. Monte Carlo-aided dosimetry of a new high dose-rate brachytherapy source. Med Phys. 1998;25(11):2200–08.

# Figure Captions

Fig. 1. 3D printing allows physicians and physicists to customize the external size and shape and the internal geometry (linear and curved catheter paths) of the brachytherapy applicator to improve treatment. Shown is a 3D printed applicator made of PC-ISO (white, top). This applicator was designed to fit a patient with a very wide vaginal canal that was too large for the largest commercial (Alpha-Omega Services, Inc.) applicator of the same type at our clinic (yellow, bottom). A cross section of the printed applicator is shown in the inset in the upper left.

Fig. 2. A set of testing apparati were designed and 3D printed for this study to measure the depth dose curve for ^192^Ir. The apparati held a piece of radiochromic film and an endobronchial brachytherapy catheter. The left picture shows the testing apparatus printed in PC-ISO, and the picture on the right shows control apparatus used to suspend a piece of radiochromic film in water. The apparati were scanned in a helical CT to compute the Hounsfield unit distribution.

Fig. 3. The test apparatus with a size 6-French endobronchial brachytherapy catheter was inserted into one of the end channels. The PC-ISO and control apparati were suspended in water before the dose was delivered.

Fig. 4. A dose plan was designed to deliver 1500 cGy at 1 cm from the center of the catheter channel in water. The dwell positions and radial dose distribution for the radiochromic film study are represented using the TG-43 dose calculation formalism in water. The films were developed for 24 hrs after exposure before they were scanned to find the dose depth curve.

Fig. 5. Shown are the distributions of Hounsfield units (HU) inside the PC-ISO apparatus (red), the Alpha Omega cylinder applicator (green), and a cup of water (blue). The mean was -1 HU for water, -10 HU for PC-ISO, and +524 for the Alpha Omega applicator. The mean HU value for the PC-ISO testing apparatus is closer to water than air (-1000 HU) or bone (+1000 HU) and more water-equivalent than the Alpha Omega cylinder.

Fig. 6. The percent dose depth from the radiochromic film test for the PC-ISO testing apparatus (red) and the control apparatus (blue). The two curves were within 1% of each other between 1 cm and 6 cm, showing that the TG-43 planning system, which assumes a water medium, can be used as normal.

Fig. 7. After the conclusion of the tests outlined in this study, we printed several GYN applicators in PC-ISO when a custom built applicator would improve treatment options. The applicators were designed specifically for each patient from measurements taken during examination and were sterilized using the STERRAD procedure before implantation and treatment. A segmented PC-ISO cylinder applicator with a custom 2.75 cm diameter is shown implanted and contoured in the patient during the dose planning process in axial (top left), sagittal (top right), and coronal (bottom left) views. The Nucletron Vaginal Tube (bright white) is 1.1 cm. Bottom right is a 3D representation of the contoured organs, applicator (blue), and tandem (purple) with the catheter visible.
